# Surging towards a better understanding of ovulation

**DOI:** 10.7554/eLife.111681

**Published:** 2026-05-18

**Authors:** Lillian Rose, Alexander S Kauffman

**Affiliations:** 1 Department of OBGYN and Reproductive Sciences, University of California San Diego La Jolla United States

**Keywords:** neuroendocrinology, kisspeptin, GnRH, LH surge, estrogen, photometry, fertility, Mouse

## Abstract

The ability to record the real-time activity of specialized neurons in the brains of female mice is providing new insights into the hormonal control of ovulation.

**Related research article** Zhou Z, Huang C-Y, Herbison AE. 2026. Prolonged oscillating preoptic area kisspeptin neuron activity underlies the preovulatory luteinizing hormone surge in mice. *eLife* **14**:RP109215. doi: 10.7554/eLife.109215.

Did you know that ovulation is controlled by a region of the brain called the hypothalamus? Rising levels of the sex hormone estrogen cause the hypothalamus to send signals to the pituitary gland, resulting in the release of a ‘surge’ of luteinizing hormone (LH). This, in turn, stimulates the ovaries to ovulate. However, the mechanisms underlying how the brain decides to initiate the LH surge remain unresolved (Goodman et al., 2022; Piet, 2023).

Neurons that make a protein called kisspeptin have an important role in this process. In the 2000s it was shown that kisspeptin neurons located in the preoptic area of the hypothalamus were sensitive to estrogen. Moreover, the activity of these kisspeptin neurons increased at the time of the LH surge, and they stimulated other neurons in the hypothalamus – GnRH neurons – that were known to trigger the LH surge (Smith et al., 2006; Robertson et al., 2009; Piet et al., 2018; Clarkson et al., 2023). Still, direct in vivo evidence of kisspeptin neurons having a role in the LH surge has been elusive.

Now, in eLife, Ziyue Zhou, Cheng-Yu Huang and Allan Herbison at the University Cambridge report that cutting-edge fiber photometry techniques have allowed them to study preoptic kisspeptin neurons in ways that were not possible before (Zhou et al., 2026). The new approach allowed the researchers to record the electrical activity of kisspeptin neurons in freely-moving female mice before, during, and after the LH surge.

Impressively, the researchers recorded continuously from the same mice for many days, across multiple stages of the estrous cycle (which is the mouse equivalent of the menstrual cycle, and typically lasts 4–5 days). This approach showed low levels of kisspeptin neuron activity each day until the proestrus stage of the cycle, when activity dramatically increased in the late afternoon.

Remarkably, the period of increased kisspeptin neuron activity lasted for about 13 hours, which was several hours longer than the LH surge itself. While the first half of the period of increased neuron activity likely caused the LH surge (Figure 1), the role of the second half remains a mystery. Zhou et al. also found that the signal from the kisspeptin neurons contained unexpected oscillations with a period of about 90 minutes. These fluctuations were present in all stages of the estrous cycle, but were most pronounced and numerous during the proestrus stage. Importantly, these oscillations – which were also shown to be dependent on estrogen – closely resemble patterns previously observed in the GnRH neurons that directly trigger the LH surge (Han et al., 2025). This supports the proposed role of preoptic kisspeptin neurons in controlling the GnRH neurons during the surge. However, at present, the physiological role of the 90 minute oscillations in both kisspeptin and GnRH neurons remains mysterious.

**Figure 1.**
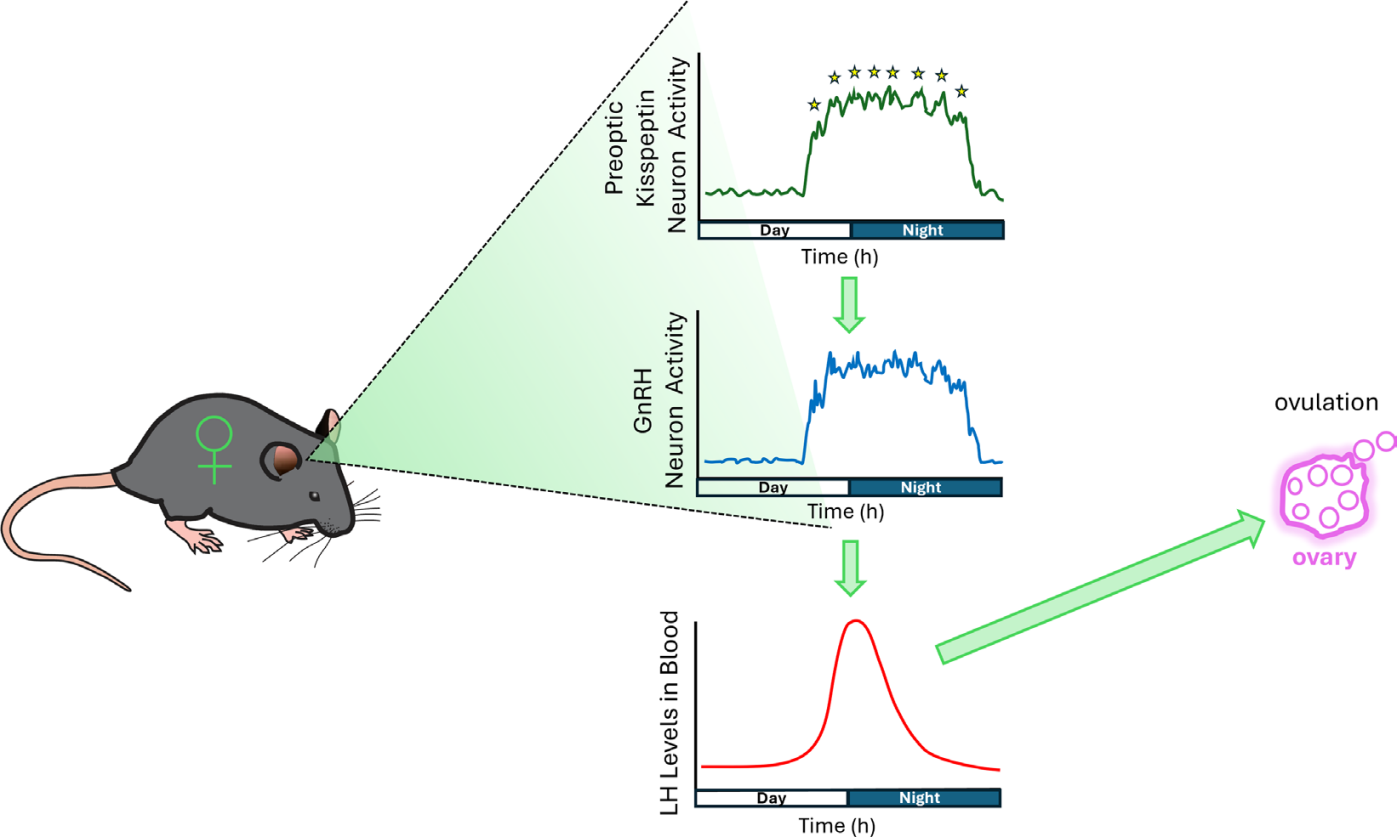
Kisspeptin neurons, GnRH neurons, and the LH surge. Top: The activity of preoptic kisspeptin neurons increases in the late afternoon of the proestrus stage of the estrous cycle, and is sustained for many hours. Oscillations with a period of 90 minutes can be clearly seen and are indicated by stars. Middle: The activity of GnRH neurons – which are downstream from the kisspeptin neurons – follows a very similar pattern (including the 90 minute oscillations), and is likely driven by the preoptic kisspeptin neurons. Bottom: The sustained firing of GnRH neurons is known to trigger the surge of luteinizing hormone (LH) to induce ovulation. Surprisingly, the LH surge (red line) ends several hours before the activity of the kisspeptin and GnRH neurons reduces. Further research is needed to understand the reasons for the 90 minute oscillations and for the extended activity of the kisspeptin neurons.

In many species, the LH surge is partly controlled by the brain’s master circadian clock (Kauffman, 2022; Piet et al., 2015; Robertson et al., 2009). Interestingly, preoptic kisspeptin neurons showed transient increases in activity each day just before nighttime, with such increases being largest on proestrus, supporting the idea that a daily circadian signal influences their function. However, unexpectedly, the timing of proestrus firing events varied both between mice and within the same mouse across cycles, suggesting kisspeptin neurons may be regulated by multiple ‘timing’ factors yet to be determined.

Might the increased kisspeptin neuron activity observed during proestrus serve roles beyond the LH surge and ovulation? Interestingly, emerging evidence suggests that kisspeptin neurons may also influence female mating behavior (Hellier et al., 2018). If so, this elevated activity on proestrus could coordinate mating with ovulation, ensuring reproductive success. This exciting possibility remains to be tested.

This new approach developed by Zhou, Huang and Herbison will greatly advance research in reproductive neuroendocrinology and could have significant clinical value. An improved understanding of how kisspeptin neurons ‘work’ in vivo could, in the future, translate into new therapeutics for reproductive disorders in women, and novel contraceptives that target select reproductive neurons in the brain.
